# Pacemaker and radiotherapy in breast cancer: is targeted intraoperative radiotherapy the answer in this setting?

**DOI:** 10.1186/1748-717X-7-128

**Published:** 2012-08-01

**Authors:** Mohammed RS Keshtgar, David J Eaton, Claire Reynolds, Katharine Pigott, Tim Davidson, Benjamin Gauter-Fleckenstein, Frederik Wenz

**Affiliations:** 1 The Breast Unit, Academic Department of Surgery, Royal Free and University College Medical School, Pond Street, London, NW3 2QG, UK; 2 Department of Radiotherapy, Royal Free Hospital, London, UK; 3 Department of Radiation Oncology, University Medical Center Mannheim, University of Heidelberg, Mannheim, Germany

**Keywords:** Pacemaker, Safety, Targeted intraoperative radiotherapy (TARGIT), INTRABEAM, Breast cancer

## Abstract

We present the case of an 83 year old woman with a cardiac pacemaker located close in distance to a subsequently diagnosed invasive ductal carcinoma of the left breast. Short range intraoperative radiotherapy was given following wide local excision and sentinel node biopsy. The challenges of using ionising radiation with pacemakers is also discussed.

## Background

Ionising radiation (IR) has been reported to interfere with modern cardiac pacemakers (PM), which are equipped with complementary metal oxide semiconductor circuitry (CMOS) [1]. In 1994, the American Association of Physicists in Medicine (AAPM) stated that a cardiac pacemaker can fail at radiotherapy doses as low as 10 Gy, and even doses of 2 Gy could lead to significant functional changes. This resulted in guidelines suggesting that the dose to the PM should be limited to 2 Gy [2].

The cardiac conditions which lead to the implantation of a PM are typically sick sinus syndrome, high grade atrio-ventricular (AV) blockade IIb, type mobitz, or total AV-blockade III. Patients suffering from high grade AV-blockade depend highly on external functional cardiac pacing since cardiac output is directly related to left ventricular pump function and heart rate. Arrhythmia results in inconstant and insufficient ventricular filling and decreased ventricular ejection fraction. This, in combination with a very low heart rate (30–50 bpm), gives rise to very low cerebral, coronary, intestinal, pulmonary and renal perfusion pressure, leading to ischemia. The patients at highest risk from pacemaker dysfunction are those who are absolutely dependent on their PM, who are without a sufficient escape rhythm. Cardiac pacemakers are programmed to sense bradycardia and to pace the heart through implanted metal coil leads which are not sufficiently shielded against radiation.

Modern PMs contain CMOS circuitry and random access memory (RAM), in addition to the battery and leads capable of sensing and pacing the heart. The CMOS is capable of signal amplification and improves device reliability and energy consumption. RAM is the programmable part of the device, holding information about patient-related anti-bradycardia pacing, detection settings and frequency thresholds. It contains a small amount of energy which is highly volatile. Some cases have been reported wherein no obvious damage to the device was found following irradiation, but the RAM had been entirely erased [3].

CMOS circuitry is built from metal-oxide-semiconductor field effect transistors. The metal oxide used in the CMOS is polycrystalline silicon (Si) and silicon dioxide (SiO_2_) is used as insulation. Energy deposition during radiotherapy using ionising radiation can result in excess electron holes in the electron valence band and electrons can leave their valence band (tunnelling). This can result in aberrant electrical pathways and reprogramming of the devices. Possible effects on the PM include altered sensitivity, amplitude changes, telemetry and programming defects (even preventing reprogramming), adjustment of function or loss of function for seconds, days or permanently.

Several cases have been reported where the threshold programming was deleted or the devices failed at low doses [1]. Therefore, in cases where the PM is close to the treatment fields for external beam radiotherapy (EBRT), adjustments may be necessary. These include modification of the field size and shape, moving the PM surgically out of the field or even withholding radiotherapy in some cases.

An alternative to EBRT for these patients might be intraoperative radiotherapy (IORT). The TARGIT trialists group has reported the result of a randomised controlled trial with this technique, which has confirmed the safety and efficacy [4].

## Case presentation

An 83 year old female patient presented with a two week history of a self detected lump in the upper outer quadrant of the left breast. Clinically there was a 15 mm suspicious lump in the left breast, mammography did not reveal any abnormality (R1) and ultrasound scan findings were consistent with the diagnosis of breast cancer (U5). Clinical and ultrasound examination of the axilla was unremarkable. Core biopsy of the lesion confirmed the diagnosis of invasive ductal carcinoma.

**Figure 1:**
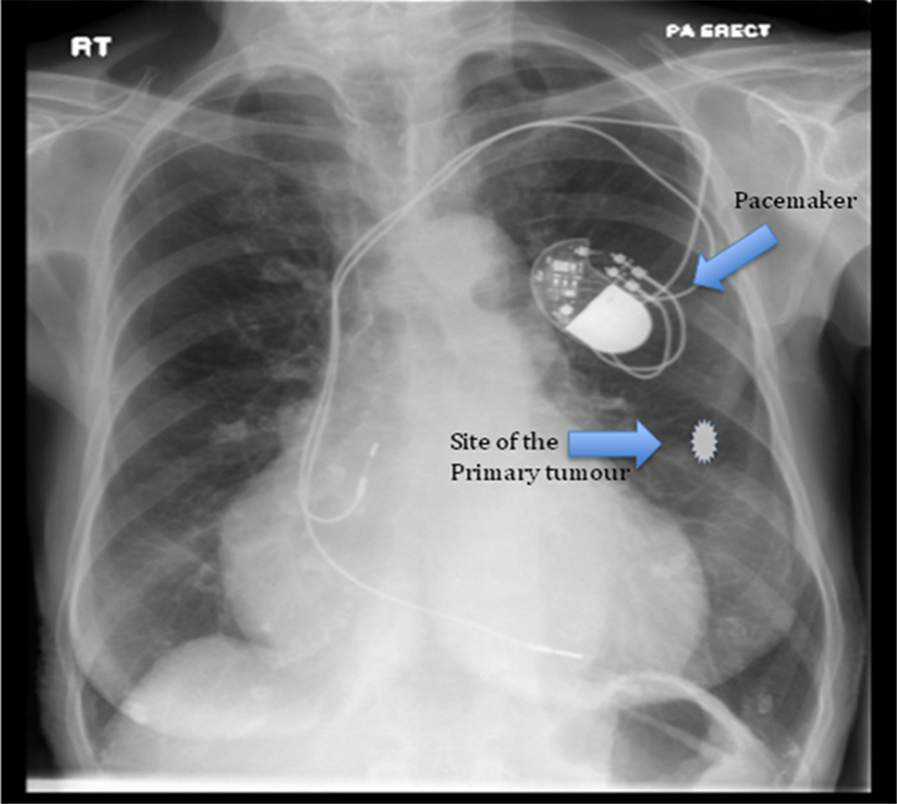
Chest x-ray of the patient showing the pacemaker in situ and the approximate position of the breast cancer.

**Figure 2:**
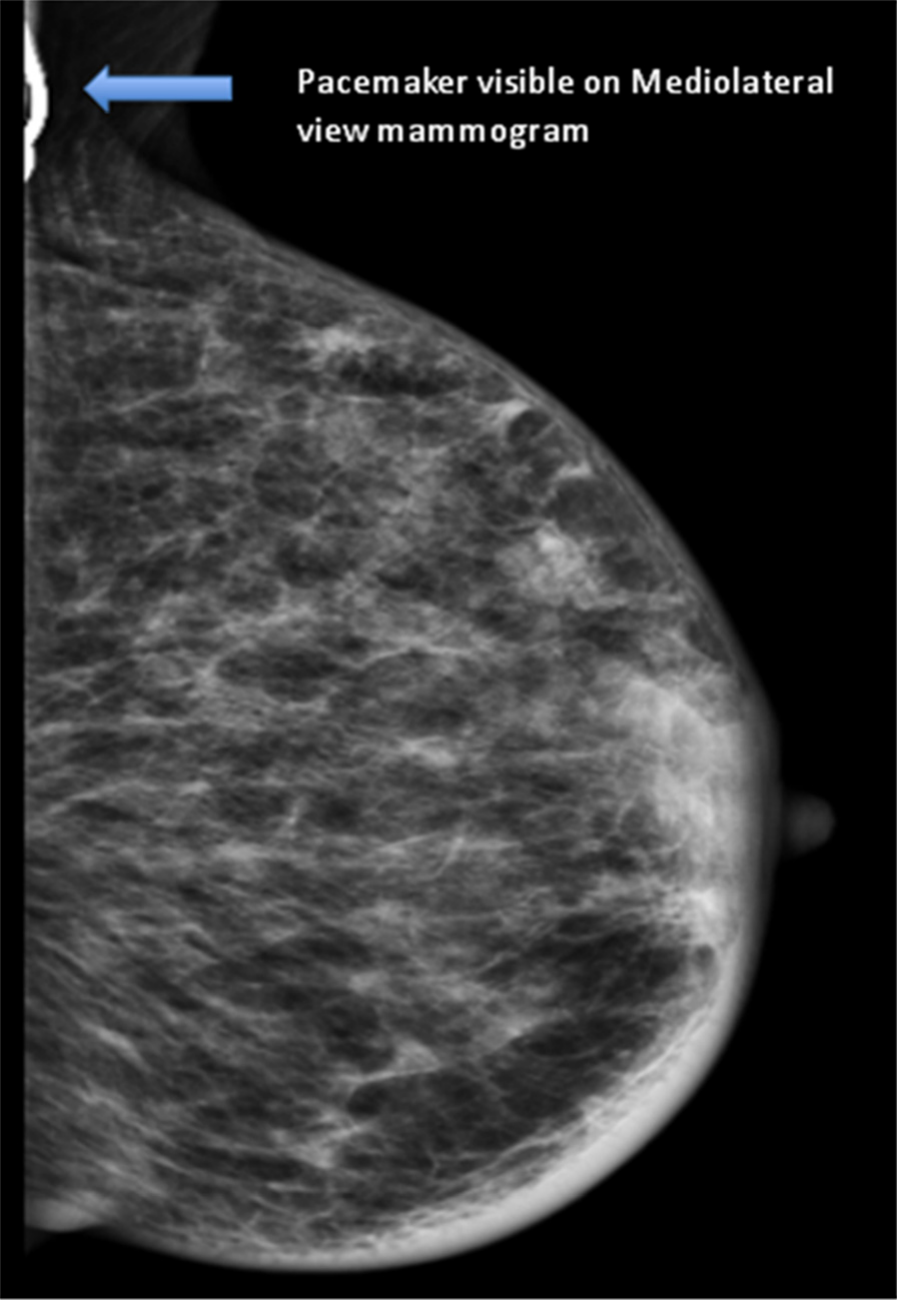
**Mammogram (mediolateral view) showing the pacemaker. **The breast cancer was mammographically occult.

During review of her past medical history, it was noted that in 1996 she had a cardiac PM inserted for persistent sinus bradycardia. In 2003 this was replaced with a St. Jude Medical dual chamber PM (St. Jude Medical Inc., St. Paul, MN, USA). The pacemaker was programmed to VVIR 70 bpm, hysterises 60 bpm in single chamber mode due to an atrial lead failure. The dominant rhythm was atrial fibrillation with intermittent ventricular pacing. The patient’s heart rate varied between 60–107 bpm. The patient had been self caring and a recent transthoracic echocardiogram showed a normal ejection fraction and left ventricular size. Anatomically, the PM was located in a subcutaneous tissue pocket in the upper pole of the left breast 9 cm away from the primary tumour (Figure 1, Figure 2).

After discussion at the multidisciplinary meeting, we recommended wide local excision and sentinel node biopsy. In view of the size of the tumour and presence of the PM, it was also decided to offer the patient intraoperative radiotherapy using the TARGIT technique.

Prior to surgery, the details of the PM were obtained from the implanting hospital and the distance from the tumour to the device was measured at an assessment session. During surgery, after harvesting the sentinel node and wide local excision, intraoperative radiotherapy was performed using the Intrabeam^™^ device (50 kV, Carl Zeiss Surgical, Oberkochen, Germany). A 3 cm diameter applicator was used, delivering approximately 20 Gy at the surface of the breast tissue in direct contact with the applicator, and 6 Gy at 1 cm from the surface, over a time of 26 minutes. During the surgery, the radiation dose to the PM was measured using thermoluminescent dosimeters (TLD), which were placed on the edge of the device closest to the x-ray source, and the distance between the applicator shaft of the Intrabeam and the PM was recorded. The measured reading was converted to dose using a batch calibration value corrected for supralinearity of dose response. The average reading of the TLD packet was 0.08 Gy.

The patient tolerated the procedure very well and there was no malfunction of the PM device during the surgery or IORT. The patient made an uneventful recovery and was discharged home the following day. The pacemaker function was tested by the cardiology team before and after treatment.

Histology confirmed the identification of a 14 mm grade 2 invasive ductal carcinoma which was completely excised. There was no lymphovascular invasion and the sentinel node was free of tumour. The cancer was ER/PR positive (quick score 8/8) and Her-2 negative. The patient was commenced on an Aromatase Inhibitor as an adjuvant treatment.

## Discussion

Although some centres treat patients in this age group with surgery and adjuvant endocrine therapy alone, within the TARGIT randomized trial there is no upper age limit for the delivery of radiotherapy. This patient would have been eligible to enter the trial were it not for the contraindication to EBRT of a pacemaker so close in distance to the tumour site. Moreover, this case is presented to highlight the issues concerning pacemakers and radiotherapy and possible approaches to overcome these problems.

As described in this case, the use of a short range kilovoltage energy x-ray source reduces the dose to normal tissues and artificial devices which are sensitive to radiation. By performing in vivo dosimetry using TLDs, we confirmed a low radiation dose to the PM. Compared to EBRT, during the IORT procedure the patient is closely monitored by an anaesthetist. Furthermore, standard monitoring such as ECG, pulse oximetry and blood pressure monitoring would help to identify any arrhythmia during the procedure. In some countries within Europe, there is a requirement for the presence of a cardiologist or a member of the cardiology team during or after IR to ensure the correct functioning of a PM. This would be reduced to a single visit for IORT in comparison to daily visits for each fraction of EBRT.

At present, little is known about the effects of direct and scattered radiation on PMs, especially the newer PMs equipped with modern CMOS circuitry. However, some manufacturers do provide estimates of the radiation doses to which their PM models can be safely irradiated (e.g. St. Jude 20–30 Gy [5], Medtronic 5 Gy [6], Guidant n.n. [7]). The wide range of these stated doses and recent in-vitro data showing that a PM can fail at any dose [8] pose significant challenges to radiation oncologists treating patients with a PM. Therefore, techniques like IORT using the Intrabeam device are an attractive alternative to existing approaches. In addition, the Intrabeam device does not produce electromagnetic interference which is a concern with linear accelerators and implantable cardioverter defibrillators [9]. Further research into the safe tolerance doses for modern PM devices will hopefully be translated into safer designs for the future. In the meantime, however, we believe that intraoperative radiotherapy using Intrabeam is a very good option for selected patients with a pacemaker.

## Conclusions

Intraoperative radiotherapy using the Intrabeam device was successfully used to treat an invasive ductal carcinoma of the left breast with a cardiac pacemaker located close in distance to the treatment area. This approach may form a viable alternative to conventional radiotherapy which has been shown to adversely effect such devices.

## Consent

Written informed consent was obtained from the patient for publication of this case report and any accompanying images. A copy of the written consent is available for review by the Editor-in-Chief of this journal.

## Abbreviations

TARGIT: Targeted intraoperative radiotherapy; IR: Ionising irradiation, CMOS, Complementary metal oxide semiconductor; PM: Pacemaker; AAPM: American Association of Physicists in Medicine; AV: Atrio-ventricular; RAM: Random access memory; EBRT: External beam radiotherapy; IORT: Intraoperative radiotherapy; TLD: Thermoluminescent dosimeter; ECG: Electrocardiogram.

## Competing interests

The authors declared that they have no competing interest.

## Authors’ contributions

MK: conception and design; MK, DE, CR, KP: patient treatment; Consultation on background and discussion of interpretation – MK, TD: surgical; KP, BG-F, FW: radiation oncology, DE: physics; MK, BG-F: drafting manuscript; DE, CR, KP, TD, FW: revising manuscript. All authors have read and approved the final manuscript.
